# UNRAVELING ALZHEIMER’S: A COMPREHENSIVE REVIEW OF BIOMARKER RESEARCH ADVANCES OVER THE PAST DECADE

**DOI:** 10.1093/geroni/igae098.2281

**Published:** 2024-12-31

**Authors:** Emily Winters, Tessa McCoy

**Affiliations:** Oregon Health & Science University, Portland, Oregon, United States; Oregon Department of Human Services, Dallas, Oregon, United States

## Abstract

Alzheimer’s Disease (AD) presents a formidable global health challenge, necessitating continuous advancements in biomarker research for enhanced diagnosis and management. This review, utilizing a methodology adapted from Arksey and O’Malley (2005) for scoping reviews, comprehensively assesses developments in AD biomarker research over the past decade. Through systematic searches of academic databases such as PubMed, Web of Science, and Scopus, articles published between 2012 and the present were analyzed, focusing on keywords encompassing AD, biomarkers, imaging techniques, blood-based markers, and international collaborative initiatives. Key findings reveal significant progress across multiple fronts of AD biomarker research, including the refinement and validation of novel imaging techniques targeting amyloid-beta and tau proteins, such as positron emission tomography (PET) imaging tracers for tau pathology. Moreover, blood-based biomarkers have emerged as promising candidates for non-invasive AD detection and monitoring, with studies investigating proteins like amyloid-beta, tau, and neurofilament light chain. The integration of longitudinal studies and multimodal imaging approaches has provided deeper insights into dynamic biomarker changes throughout disease progression, while machine learning and data analytics have identified novel biomarker patterns and predictive models, enhancing diagnostic accuracy and personalized treatment strategies. International collaborative efforts, exemplified by initiatives like the Alzheimer’s Disease Neuroimaging Initiative (ADNI) and the Dominantly Inherited Alzheimer Network (DIAN), have fostered data sharing and large-scale biomarker studies, accelerating the pace of discovery and validation. In conclusion, this comprehensive assessment underscores significant strides in AD biomarker research, promising improved early detection, accurate diagnosis, and targeted therapeutic interventions, ultimately advancing patient outcomes and disease management.

